# Computational pathology aids derivation of microRNA biomarker signals from Cytosponge samples

**DOI:** 10.1016/j.ebiom.2022.103814

**Published:** 2022-01-17

**Authors:** Neus Masqué-Soler, Marcel Gehrung, Cassandra Kosmidou, Xiaodun Li, Izzuddin Diwan, Conor Rafferty, Elnaz Atabakhsh, Florian Markowetz, Rebecca C. Fitzgerald

**Affiliations:** a MRC Cancer Unit, Box 197, Cambridge Biomedical Campus, Cambridge, CB2 0XZ, UK; b Cancer Research UK Cambridge Institute, Li Ka Shing Centre, Robinson Way, Cambridge, CB2 0RE, UK; c Abcam Inc., 1 Kendall Sq B2304, Cambridge, MA, 02139, United States

**Keywords:** Barrett's oesophagus, Oesophageal cancer, Computerized image analysis, Artificial intelligence, Dysplasia, Screening

## Abstract

**Background:**

Non-endoscopic cell collection devices combined with biomarkers can detect Barrett's intestinal metaplasia and early oesophageal cancer. However, assays performed on multi-cellular samples lose information about the cell source of the biomarker signal. This cross-sectional study examines whether a bespoke artificial intelligence-based computational pathology tool could ascertain the cellular origin of microRNA biomarkers, to inform interpretation of the disease pathology, and confirm biomarker validity.

**Methods:**

The microRNA expression profiles of 110 targets were assessed with a custom multiplexed panel in a cohort of 117 individuals with reflux that took a Cytosponge test. A computational pathology tool quantified the amount of columnar epithelium present in pathology slides, and results were correlated with microRNA signals. An independent cohort of 139 Cytosponges, each from an individual patient, was used to validate the findings via qPCR.

**Findings:**

Seventeen microRNAs are upregulated in BE compared to healthy squamous epithelia, of which 13 remain upregulated in dysplasia. A pathway enrichment analysis confirmed association to neoplastic and cell cycle regulation processes. Ten microRNAs positively correlated with columnar epithelium content, with miRNA-192–5p and -194–5p accurately detecting the presence of gastric cells (AUC 0.97 and 0.95). In contrast, miR-196a-5p is confirmed as a specific BE marker.

**Interpretation:**

Computational pathology tools aid accurate cellular attribution of molecular signals. This innovative design with multiplex microRNA coupled with artificial intelligence has led to discovery of a quality control metric suitable for large scale application of the Cytosponge. Similar approaches could aid optimal interpretation of biomarkers for clinical use.

**Funding:**

Funded by the NIHR Cambridge Biomedical Research Centre, the Medical Research Council, the Rosetrees and Stoneygate Trusts, and CRUK core grants.

**Research in Context**

**Evidence before this study**

Several microRNAs (miRNA) play a role in pre- and neoplastic processes, including Barrett's oesophagus and oesophageal cancer. However, the cell attribution of these biomarkers is not clear in cytology sampling methods such as Cytosponge, which is a non-endoscopic pan-oesophageal sampling method that can contain columnar epithelium from the gastric cardia as well as Barrett's metaplasia.

Computational pathology tools and artificial intelligence are leading to improvement of diagnostic methods and refinement of biomarker detection.

**Added value of this study**

Being able to perform a quality control of cytology samples is an important metric to ensure successful sampling. This study uses a computational pathology method and multiplex miRNA analysis. By using orthogonal methods we are able to evaluate the origin of miRNA expression profiles from Cytosponge samples, which has not been done before.

**Implications of all the available evidence**

We found 23 miRNAs that are differentially regulated in diseased versus control samples. The use of computational pathology enabled us to reliably quantify the amount of columnar epithelium in each sample so it could be correlated with miRNA expression levels. This elucidated two gastric-specific miRNAs that determine whether the gastric cardia was successfully sampled, and a set of miRNAs biomarkers for Barrett's oesophagus. miRNA analyses have promise for use with Cytosponge and future work is required to test this method prospectively. Furthermore, this combined methodology could be applied to other biomarker studies to determine the cellular origin of the signal.

Alt-text: Unlabelled box

## Introduction

Biomarkers are critical for early detection of cancer that cannot otherwise be easily and reliably detected from morphological analysis of fluid, biopsy or cytology samples. To facilitate high data throughput, bulk assays are often performed on entire cell samples, yielding no spatial or cellular tissue information. New methods such as single cell sequencing are improving experimental resolution, but they are not feasible for large-scale clinical application and spatial features are not retained from single cell analysis.

Non-endoscopic cell collection tools coupled with novel biomarkers have been developed as an alternative to endoscopy for the early detection of oesophageal cancer.^1^, ^2^, ^3^, ^4^, ^5^, ^6^ These tools are promising to identify patients with risk factors such as heartburn symptoms who have the premalignant condition Barrett's oesophagus (BE) who warrant endoscopy. Our group has developed a the Cytopsonge-TFF3 test, which is a non-endoscopic, pan-oesophageal collection device that is coupled with pathological assessment and immunohistochemical staining for protein TFF3 to identify pathognomonic goblet cells.^7^, ^8^, ^9^, ^10^ We have recently shown that an offer of the Cytosponge-TFF3 test in primary care increases the detection rate for BE by 10-fold (10.6, 95% CI: 6.0–18.8, *p* = 0.0004) when compared to standard of care.^11^

A technical challenge for analysis of the Cytosponge sample is the high proportion of normal squamous cells that reduce the relative cellularity of the Barrett's cells of interest. Depending on the length of the BE segment the cells containing intestinal metaplasia may be as sparse as 1% or less. Therefore, any biomarker applied to the sample has to be highly sensitive. Furthermore, if the Cytosponge device does not reach the stomach, the distal oesophagus may not be adequately sampled. For this reason, samples with fewer than five columnar groups are reported as a *low confidence* or *inadequate* samples.^12^ Hence, the BEST2 trial reported an overall sensitivity of 79.9% in a per protocol analysis that included inadequate samples.^13^ By comparison, when inadequate samples were excluded, the sensitivity was uniformly high across the participating centres (91–98%).^8^ In the subsequent BEST3 trial, patients with a low confidence result were invited for a repeat test to increase the sensitivity and confidence in the result.^11^

As an alternative to the immunohistochemical biomarker TFF3, it is worth considering molecular-based assays that could be more readily scaled up and reported in a more quantitative fashion. This would ideally need to include a marker of gastric cells to ensure adequacy of the sample. Our group therefore examined whether other molecular biomarkers such as microRNAs (miRNA or miRs) or changes in methylation status could be used to identify BE.^14^^,^^15^ We previously identified 15 miRNAs that were up-regulated in BE vs squamous oesophageal (NE) tissues, and a 6-plex panel was shown to have an AUC of 0.89 to detect BE cases with 86.2% sensitivity and 91.6% specificity. When adequate samples were included for analysis, the panel had a comparable accuracy to the IHC-based Cytosponge-TFF3 test performed in the same sample set (AUC 0.89).^14^

Because the whole cytology sample is processed for these liquid assays including for miRNAs, it is not possible to determine the cellular source of the differential signal. The signature observed in a bulk sample may be related both to gastric cardia (GC) as well as to Barrett's columnar cells,^16^^,^^17^ as several miRNAs have been shown to be upregulated in GC compared to normal squamous epithelium (NE).^18^ Thus, gastric columnar cells could be helpful to determine sample adequacy but may lead to confounding. The challenge therefore remains to correlate the miRNAs with different cell types and to not only diagnose Barrett's but also to assess for dysplasia. This is important since only 0.3% patients per year will progress to adenocarcinoma.^19^^,^^20^ The risk of cancer progression increases significantly once low or high grade dysplasia occurs and given the poor outcomes for invasive disease, early detection and intervention is needed.^21^

We hypothesized that combining computational pathology to quantify the columnar epithelium (CE) present in the H&E sample together with a novel quantitative miRNA assay would enable us to differentiate samples with GC material and determine the extent of intestinal metaplasia and dysplasia to aid in clinical decision making at scale. A new assay called FirePlex™ provides custom multiplex panels for miRNA profiling, which are applicable to miRNA from FFPE material and have the ability for high throughput application. Thus, we performed a 110-plex miRNA expression analysis and computational pathology across a test cohort of 117 FFPE Cytosponge samples across different disease states and the findings were confirmed in an independent cohort of a further 139 Cytosponge samples.

## Methods

### Ethics

All samples originated from the BEST2 study, with ethics approval obtained from the East of England–Cambridge Central Research Ethics Committee (No: 10/H0308/71). All patients consented their Cytosponge samples for research use.

### Study design

**Figure 1:**
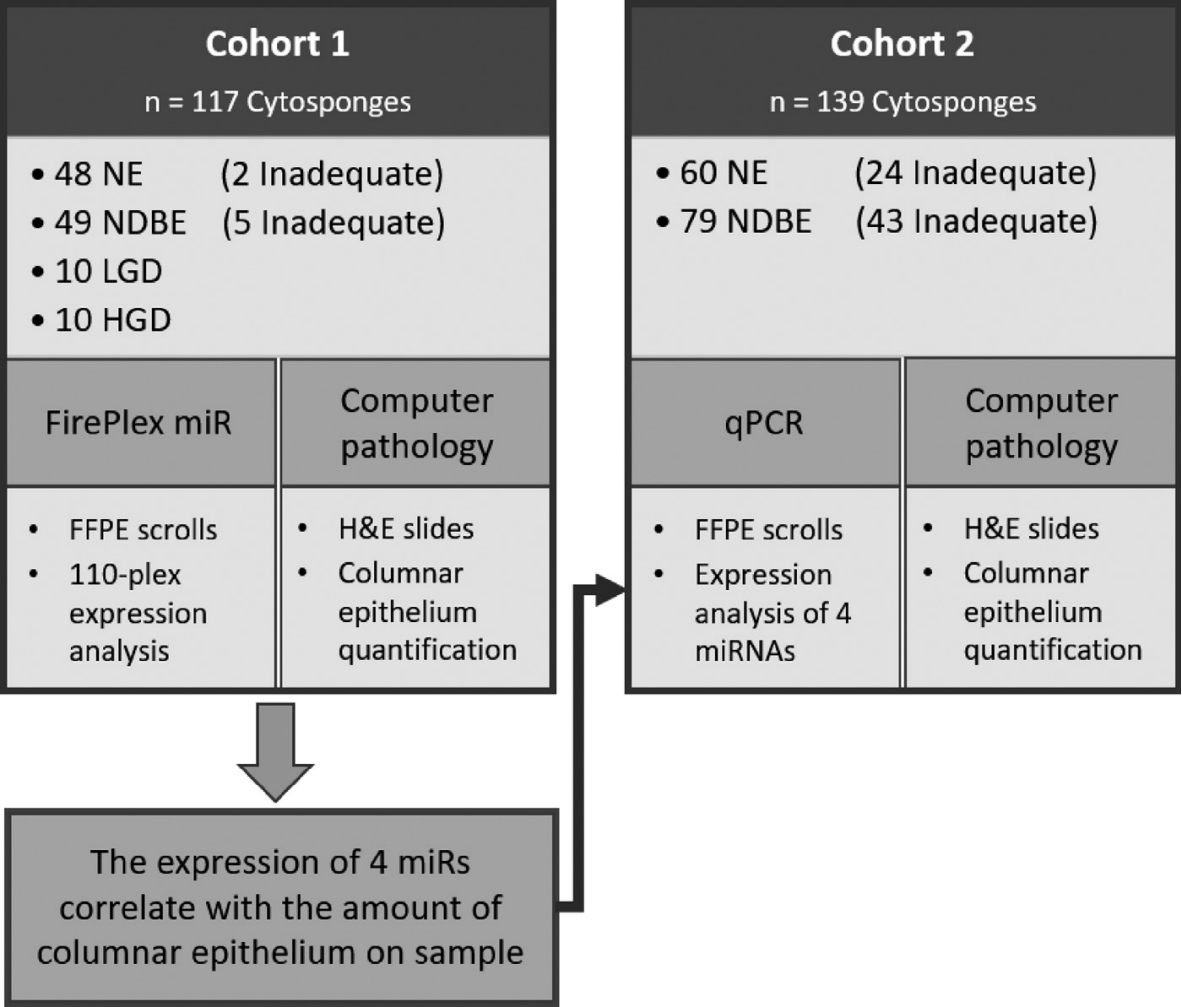
Study design. NE: normal epithelium, NDBE: non-dysplastic Barrett's oesophagus, LGD: low-grade dysplasia, HGD: high-grade dysplasia, FFPE: formalin-fixed paraffin-embedded, H&E: haematoxylin and eosin, qPCR: quantitative PCR. Inadequate refers to samples containing no columnar material suggesting that the capsule did not reach the stomach.

This observational, cross-sectional study was comprised of two cohorts: The first contained 117 samples, including 48 NE, 49 non-dysplastic BE (NDBE), 10 LGD, and 10 HGD samples, as summarized in Table 1 and Figure 1, and was designed to explore the sensitivity of the FirePlex technology with a custom-designed panel in Cytosponge samples.

**Table 1:** Demographic data of Cohorts 1 and 2. BMI: Body mass index. M: Maximum height of the Barrett's lesion. Ns: non-significant. NDBE: non-dysplastic BE. DBE: dysplastic BE. Numbers show events except for the biometric variables of age, BMI, and waist-to-hip ratio, where mean values are represented. Percentages are shown in parenthesis, except for standard deviation in the biometric variables. Chi-squared (Χ^2^) was performed for gender, smoking, alcohol, and aspirin use. For age, BMI, and waist-to-hip ratio a Kruskal-Wallis test was chosen, while for the BE lesion variable, a one-way ANOVA was performed and t-tests for 2-group comparisons.

	COHORT 1				COHORT 2				
	NE	NDBE	DBE	TOTAL	Adequates		Inadequates		TOTAL
					NE	NDBE	NE	NDBE	
Gender **– no. (%)**									
** Males**	22 (45.8)	40 (81.6)	19 (95)	81 (69.2)	18 (50)	27 (75)	12 (50)	34 (79.1)	91 (65.5)
** Females**	26 (54.2)	9 (18.4)	1 (5)	36 (30.8)	18 (50)	9 (25)	12 (50)	9 (20.9)	48 (34.5)
Age median **– years (SD)**	49.1 (45.2–52.9)	64.5 (47.7–81.3)	67.2 (62–73.4)	58.5 (55.8–61.2)	55.9 (51.1–60.6)	63.7 (53.2–74.3)	63 (57.2–68.9)	64.2 (60.4–67.9)	61.7 (59.4–64)
BMI median **– index (SD)**	28.5 (27.1–29.9)	28.5 (27.3–29.6)	28.6 (27.3–30.3)	28.8 (27.6–29.5)	27.1 (25.4–28.9)	28.6 (26.9–30.2)	28.3 (26.3–30.3)	28.5 (27.1–30)	28.1 (27.3–29)
Waist/hip median **– ratio (SD)**	0.89 (0.86–0.91)	0.94 (0.92–0.96)	0.97 (0.95–1)	0.93 (0.91–0.94)	0.89 (0.86–0.92)	0.94 (0.90–0.98)	0.95 (0.86–1.04)	0.95 (0.93–0.98)	0.93 (0.91–0.95)
Longest BE lesion **– no. (%)**									
**M< 3**	0	10 (20.4)	3 (15)	13 (11.1)	0	7 (19.4)	0	10 (23.25)	17 (12.2)
**M ≥ 3**	0	35 (71.4)	14 (70)	49 (41.9)	0	18 (50)	0	23 (53.5)	41 (29.5)
** M n/a**	48 (100)	4 (8.2)	3 (15)	55 (47)	36 (100)	11 (30.6)	24 (100)	10 (23.25)	81 (58.3)
Smoker status **– no. (%)**									
** Anytime**	27 (56.3)	28 (57.1)	15 (75)	70 (59.8)	20 (55.6)	22 (61.1)	14 (58.3)	27 (62.8)	83 (59.7)
** Never**	20 (41.7)	21 (42.9)	5 (25)	46 (39.3)	16 (44.4)	14 (38.9)	10 (41.7)	16 (37.2)	56 (40.3)
** N/a**	1 (2.1)	0	0	1 (0.9)	0	0	0	0	0
Alcohol intake **– no. (%)**									
** Never**	11 (22.9)	11 (22.4)	4 (20)	26 (22.2)	9 (25)	8 (22.2)	8 (33.3)	7 (16.3)	32 (23)
** Occasional**	13 (27.1)	10 (20.4)	5 (25)	28 (23.9)	10 (27.8)	4 (11.1)	7 (29.2)	12 (27.9)	33 (23.7)
** Weekly**	16 (33.3)	18 (36.7)	7 (35)	41 (35)	11 (30.6)	13 (36.1)	4 (16.7)	13 (30.2)	41 (29.5)
** Daily**	7 (14.6)	10 (20.4)	4 (20)	21 (17.9)	6 (16.7)	11 (30.6)	5 (20.8)	11 (25.6)	33 (23.7)
** N/a**	1 (2.1)	0	0	1 (0.9)	0	0	0	0	0
Total	**48**	**49**	**20**	117	**36**	**36**	**24**	**43**	139

The second cohort consisted of 139 samples, with 72 adequate (36 NE and 36 NDBE) samples and 67 inadequate (24 NE and 43 NDBE) samples to assess the importance of GC cellularity content in miRNA expression. We purposefully enriched this second cohort for samples that were classed as inadequate. Those i*nadequate* samples were defined as having less or equal than five columnar cell groups from GC. The highest pathology grade following gold-standard endoscopy with Seattle protocol biopsies was taken as the sample's diagnosis label. Each Cytosponge sample was independent, originating from an individual patient.

### Cytosponge specimen and scrolls

FFPE scrolls were obtained from previously collected Cytosponge blocks as previously described.^8^^,^^9^ Scrolls (two for Cohort 1 and four for Cohort 2) of 6μm thickness were cut for RNA extraction kept at 4 °C until processed. Two adjacent 3.5μm scrolls were used for haematoxylin and eosin (H&E) staining. Cohort 1 was processed using the FirePlex™ platform and Cohort 2 was analysed using a pre-specified qPCR assay for a small miRNA panel determined from the FirePlex™ results.

### Multiplex miRNA expression analysis

A custom panel of 110 miRNA target molecules was used with Abcam's FirePlex™ platform (Abcam, Cambridge, UK). The miRNA targets for the bespoke panel were chosen based on results from previous studies and following a literature review of known miRNA profiles in BE and OAC (Supplementary data ST1). FirePlex™ uses hydrogel particles that specifically capture amplified miRNA products and can be quantified by flow cytometry.^22^ Each custom panel can contain up to 70 targets, and therefore two panels (A and B), were constructed each containing 55 miRNA-specific hydrogel particles, as well as controls (three negative controls, 10 normalizer particles, and two assay control particles), making a total of 70 distinct particles. The miRNA of Cohort 1 was extracted using a Lysis Mix made with Protease Mix and Digest Buffer from FirePlex™’s miRNA Assay kit V2 according to the manufacturer's instructions (see Supplementary Methods). Results were read using flow cytometers LSRII (BD Biosciences, USA) and Guava (Luminex, USA).

### Histopathological analysis with a computational pathology tool

**Figure 2:**
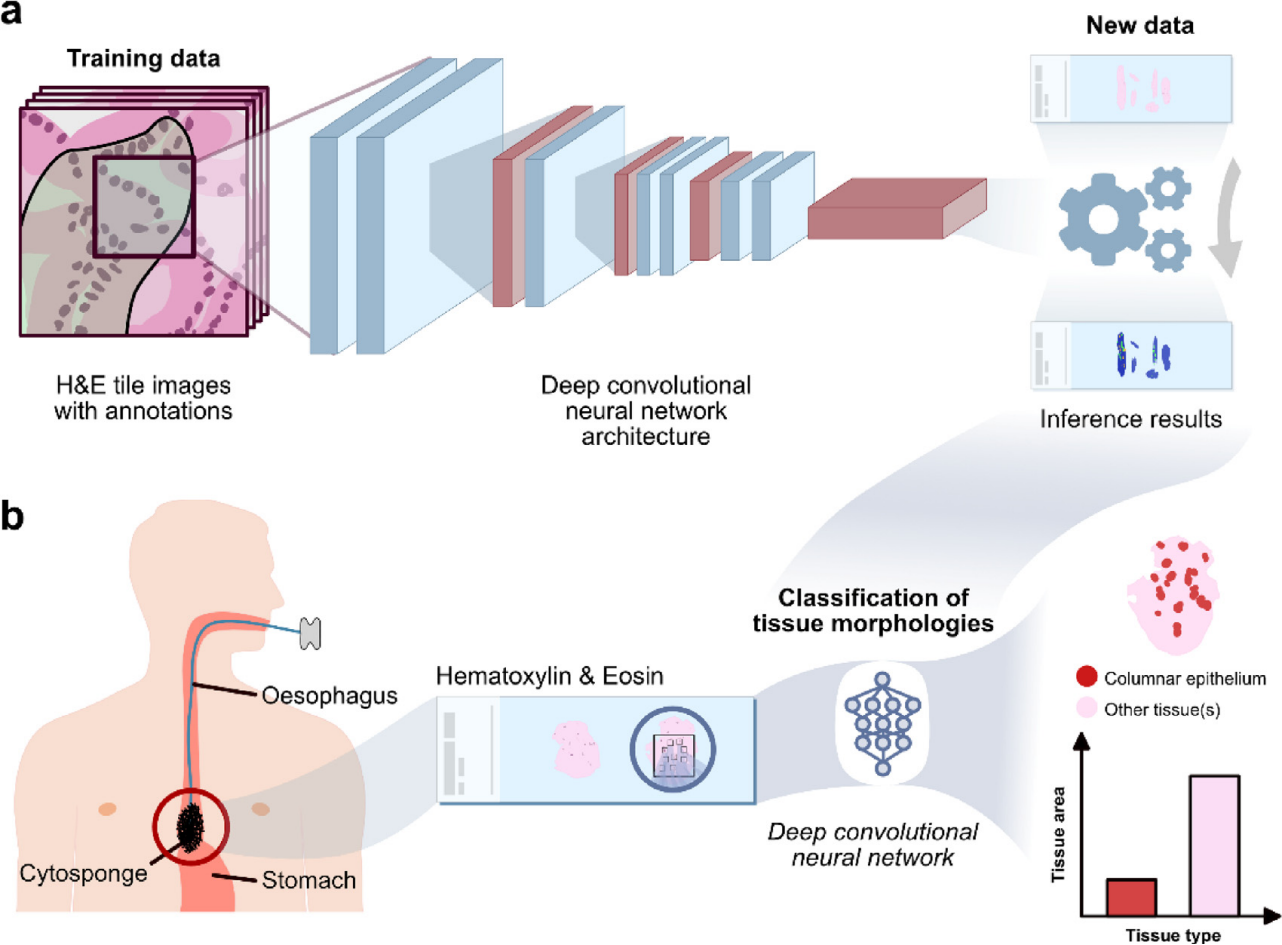
**a**. Schematic of the computational pathology training using a deep convolutional neural network architecture. **b.** Methodology to quantify the amount of CE in H&E slides of Cytosponge samples.

The H&E slides were scanned on an Aperio AT2 digital whole-slide scanner (Leica Biosystems Nussloch GmbH, Germany) at 40x magnification. Deep learning models were trained and validated on multiple datasets^23^ (Figure 2). In brief, tiles with a size of 400 by 400 pixels (corresponding to 200 by 200 pixels at a magnification of 40x) were extracted from regions with tissue (determined by Otsu thresholding) and passed through a convolutional neural network (i.e. VGG-16) to determine the cell/tissue type. After inference was completed for one whole-slide image, aggregation of tiles was performed by using pre-determined thresholds.^23^ All tiles containing CE (of any type except respiratory type) were counted and used for comparison with miRNA expression values (Figure 2).

### miRNA extraction and quantitative PCR (qPCR)

In Cohort 2, four scrolls were used for miRNA extraction with the miRNeasy Mini kit (#217004, QIAgen, Hilden, Germany) by following the manufacturer's protocol.

For the reverse transcription (RT-PCR) reaction, miScript II RT kit (#218160, QIAgen) and miScript HiSpec buffer were used. Once the RT-PCR was performed, the product was used in a 1:10 dilution on the quantitative PCR (qPCR) on a Light Cycler 480 II (Roche Diagnostics, Switzerland). The QuantiNova SYBR green PCR kit and the universal reverse primer from miScript SYBR green kit (#208052 and #218073 both from QIAgen) were used for the qPCR. The specific forward primers, which were used at 10 uM are listed in Supplementary Table ST5. The cycling reaction started at 95 °C for 15 min, followed by 45 cycles of amplification steps of 95 °C, 60 °C and 72 °C for 10 s each, and a melting curve of 95 °C for 5 s, 65 °C for 1 min and 97 °C, finished with a cooling step. Hsa-miR-103 and −191 were used for normalization consistent with previously published microarray data.^14^ Only cases with data from all four test miRs were considered.

### Statistical analysis

A preliminary analysis of the data output was performed in FirePlex™’s Analysis Workbench provided by Abcam. The software was used to merge and adjust signal values from Panel A and B via the 10 common endogenous targets present in both panels and as well as a background subtraction. The geNorm function was applied to the dataset by using the three most stable miRNA targets. Seven samples with too low signals were not normalized and thus discarded for analysis. There was minimal clinical data missing, which was not imputed.

Two key objectives were pursued through statistical analysis in this work: first, the comparison and differential expression of different miRNAs between disease states in Cohort 1 and 2. And second, the correlation of miRNA expressions and cell type classifications extracted via computational pathology, which uses scanned pathological slides to generate tissue type labels via machine learning methods. Data analysis and plotting was performed with the SciPy and scikit-learn libraries in Python. Statistical analyses/tests used were: ANOVA, unpaired *t*-test, Mann-Whitney (for two groups) or Kruskall-Wallis (for ≥3 groups) tests, Receiver Operating Characteristics (ROC) for the accuracy of the miRNAs expression in detecting columnar epithelium. In particular, the 95% confidence intervals were produced by bootstrapping with replacement at equal sample size. Spearman ρ correlation was used to evaluate the relationship between miRNA expression levels and columnar epithelium content in the sample. Statistical significance was assumed at *p* ≤ 0.05. Bonferroni correction as a post-hoc test was used for all multiple comparisons. When needed, univariate analyses were performed prior to multivariate analyses. Box plot whiskers show 5th to 95th percentile data points. Statistical significance is shown with asterisks, * representing *p* ≤ 0.05, ** *p* ≤ 0.01, *** *p* ≤ 0.001 and **** *p* ≤ 0.0001 in t-tests.

### Pathway and mRNA binding analysis by top miRs

mRNA targets for the top miRNA targets were retrieved from the target prediction tool miRabel.^24^ Targets with recorded experimental validation were selected preferentially. The top 100 mRNA targets with *a* <0.05 miRabel score for each miR were fed to the STRING database (version 11.0b). The STRING database (https://string-db.org/)^25^ was used to explore the pathways where the mRNA bound by our miRNA of interest were implicated, with the strength of the biological process or molecular function predictions being considered (see Supplementary data for details). The miRbase (http://www.mirbase.org/)^26^ database was queried for basic miRNA legacy information.

### Role of funding source and patient involvement

None of the funders were directly involved in the design, data collection, analysis, or interpretation, or writing of this report. Patient involvement was achieved during the BEST2 study^13^ and we plan to disseminate the manuscript results to the wider public through social networks and fair events in person when possible.

## Results

### Differential miRNA expression between Cytosponges from healthy and diseased oesophagus

The two cohorts selected for this study reflect the known demographics of BE. As expected, there was a bias towards male sex, abdominal fat (waist-to-hip ratio) and older age in Barrett's patients (with or without dysplasia), and these factors were statistically significantly higher in BE compared to NE groups in our two study cohorts (Table 1 and Supplementary figures S1 and S2). Supplementary figures S1 and 2 have more information for inter-group comparisons and show that cohorts 1 and 2 ultimately are comparable in terms of overall clinical demographics.

The expression of 110 miRNA targets was profiled across 117 FFPE Cytosponge samples from cohort 1 using the FirePlex™ platform (Abcam, Cambridge, UK). This cohort was formed of NE, NDBE, LGD and HGD samples (Table 1). A total of four samples were excluded from normalization and analysis due to low signal (1 NE, 2 NDBE, and 1 HGD). A further seven cases were excluded from initial analysis as they had not reached the stomach upon sampling (≤5 gastric gland groups in diagnostic H&E slides). These *inadequate* samples included five NDBE patients and two with NE.

**Figure 3:**
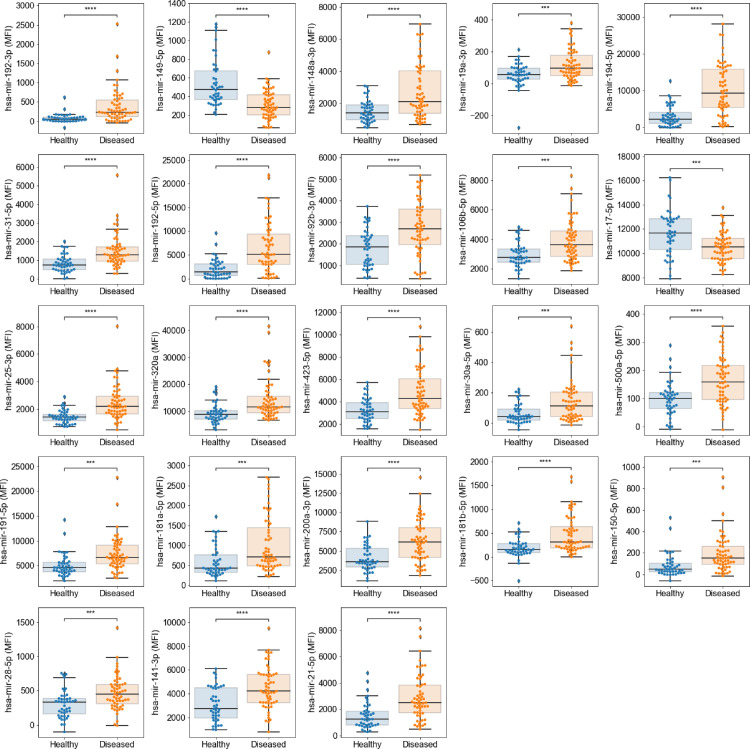
23 miRNAs with significantly differential expression (*p*<0.05 [Mann-Whitney]) between healthy and diseased Cytosponge samples. Only miR-149–5p and miR-17–5p were lower in diseased (*n* = 67) than in healthy (*n* = 46) samples.

When samples were grouped into healthy (NE, *n* = 43) and diseased (NDBE, LGD, and HGD; *n* = 61) tissues, a Mann-Whitney test with Bonferroni correction showed a total of 23 miRNAs with significant differential expression (Figure 3). For the vast majority of miRNAs, their expression in healthy tissue was lower than in diseased samples.

### miRNA markers for Barrett's oesophagus in Cytosponge samples

**Figure 4:**
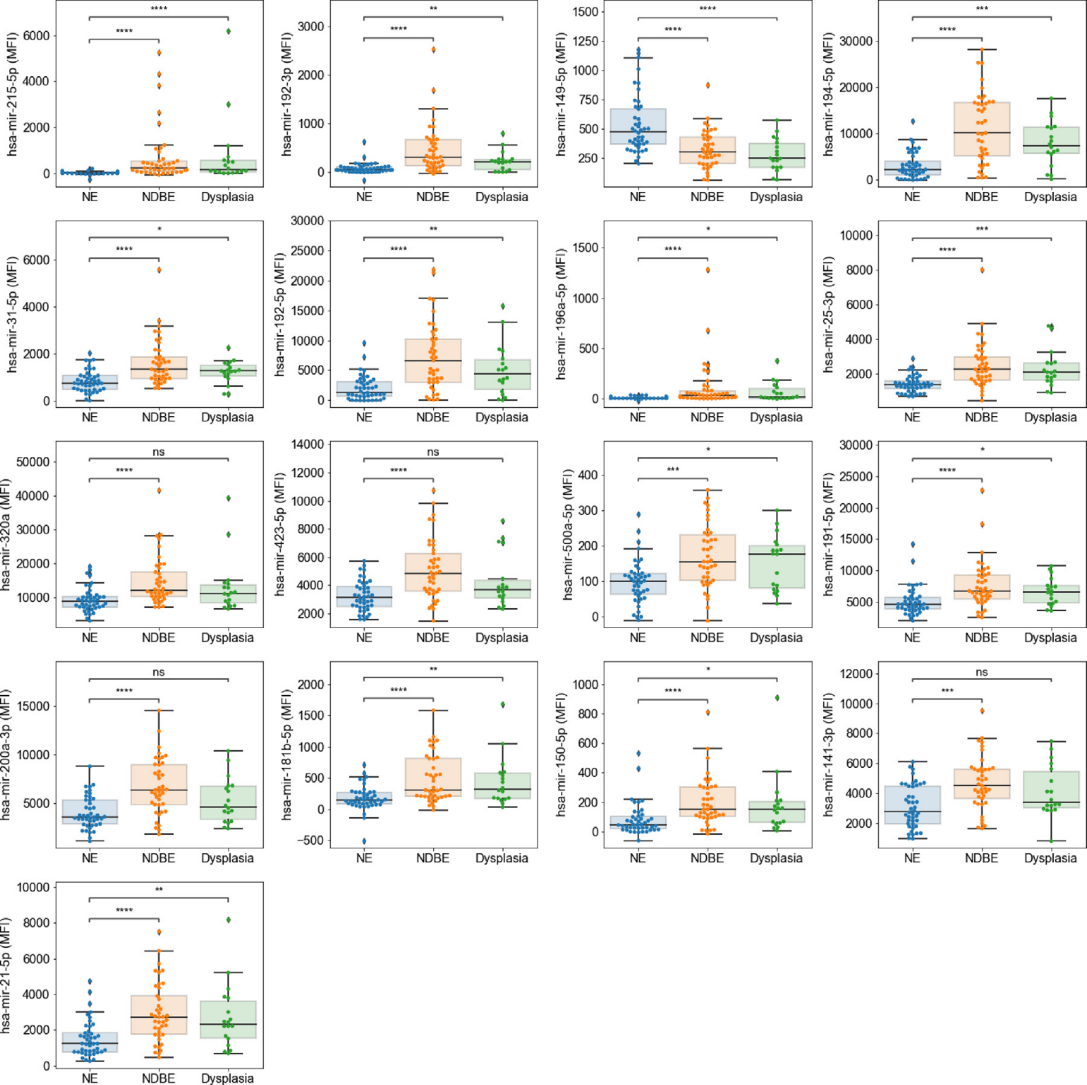
Top 17 markers with differential expression (*p*<0.05 [Kruskall-Wallis]) in NE (*n* = 46) versus NDBE (*n* = 47) or dysplasia (*n* = 20) groups.

Next, we were interested in comparing miRNA expression in dysplastic and non-dysplastic BE samples when compared to NE, using Kruskal-Wallis tests with Bonferroni correction, with a total of 17 markers being selected for upregulation in either NDBE or dysplasia (Figure 4).

While none of the 110 markers analysed were upregulated exclusively in the dysplasia group, 13 miRs remained upregulated in NDBE and dysplastic cases compared to the NE group. These were miR-21–5p, −25–3p, −31–5p, −149–5p, −150–5p, −181b-5p, −191–5p, −192–3p, −192–5p, −194–5p, −196a-5p, −215–5p and −500a-5p.

### Pathway analysis of downstream mRNA targets of shortlisted miRs

In order to examine the underlying biology, 17 miRNAs were queried using the STRING database due to their upregulation in NDBE, high and low grade dysplasia compared to NE Cytosponges (Figure 4). We thus queried miR-21–5p, −25–3p, −31–5p, −141–3p, −149–5p, −150–5p, −181b-5p, −191–5p, −192–3p, −192–5p, −194–5p, −196a-5p, −200a-3p, −215–5p, −320a, −423–5p, and −500a-5p which returned 7217, 8623, 9438, 8729, 11,193, 11,451, 9919, 5142, 7689, 8008, 8222, 8248, 8677, 7909, 10,240, 10,031 and 8758 mRNA targets, respectively, which were queried in STRING.

While miR-196a-5p had a strong association to pathways related to glandular epithelium phenotypes, most of the remaining miRNAs were associated with cell division and cancerous process pathways, such as p53 signalling and cellular senescence. Table 2 summarizes the relevant pathway enrichments found.

**Table 2:** STRING database result summary of BE-related miRNAs. Nonsignificant results are marked with ns. KEGG pathway involvement is marked with^#^.

miRNA	Pathway enrichment or KEGG pathway	Strength
21–5p	Regulation of metalloendopeptidase activity	1.99
	Fatty acid elongation	1.75
25–3p	Sterol regulatory element-binding protein signalling pathway	2.29
	miRNA in cancer^#^	0.9
31–5p	ns	–
141–3p	Regulation of oxidative stress-induced intrinsic apoptotic signalling pathway	2.04
	miRNA in cancer^#^	1.22
	Cellular senescence^#^	0.84
149–5p	ns	–
150–5p	Regulation of muscle cell differentiation	1.02
181b-5p	Regulation of endodeoxyribonuclease activity	1.69
	Nuclear-transcribed and histone mRNA catabolic process	1.69
	Intrinsic apoptotic signalling pathway in response to oxidation	1.54
	miRNA in cancer^#^	0.96
	Cellular senescence^#^	0.94
191–5p	ns	–
192–3p	ns	–
192–5p	Protein kinase binding	0.51
	Adenyl ribonucleotide binding	0.5
194–5p	Negative regulation of nitric oxide biosynthetic process	1.81
	Inactivation of MAPK activity	1.68
	Epithelial cell signalling in *Helicobacter pylori* infection^#^	1.28
196a-5p	Lung secretory cell differentiation	2.64
	Intestinal epithelial cell differentiation	2.34
	Glandular epithelial cell differentiation	2.01
200a-3p	Positive regulation of cardioblast differentiation	2.07
	miRNA in cancer^#^	1.25
	p53 signalling pathway^#^	1.24
215–5p	DNA replication initiation	1.4
	G1/S cell cycle phase transition	1.1
320a	Negative regulation of peptidyl‑serine dephosphorylation	2.12
	Shigellosis^#^	1.09
	miRNA in cancer^#^	0.9
	Cellular senescence^#^	0.8
423–5p	ns	–
500a-5p	ns	–

### Differential miRNA expression across all sample types suggests a columnar phenotype as the tissue of origin

To explore the strength of the markers detected in the healthy/diseased and dysplasia-specific analysis, we performed an analysis that shortlisted 14 miRs having differential expression with statistical significance between NE, NDBE, LGD or HGD (Supplementary figure S3).

The analysis showed that four miRNAs were virtually undetectable in inadequate cases: miR-192–3p, −192–5p, −194–5p, −196a-5p. The same miRs showed a median fold change of ≥2 in NDBE, LGD or HGD compared to NE samples (Supplementary table ST4). This suggested that the differential miRNA signal might be originating from columnar cells, including gastric cardia cells sampled by the Cytosponge, and not only BE cells.

**Figure 5:**
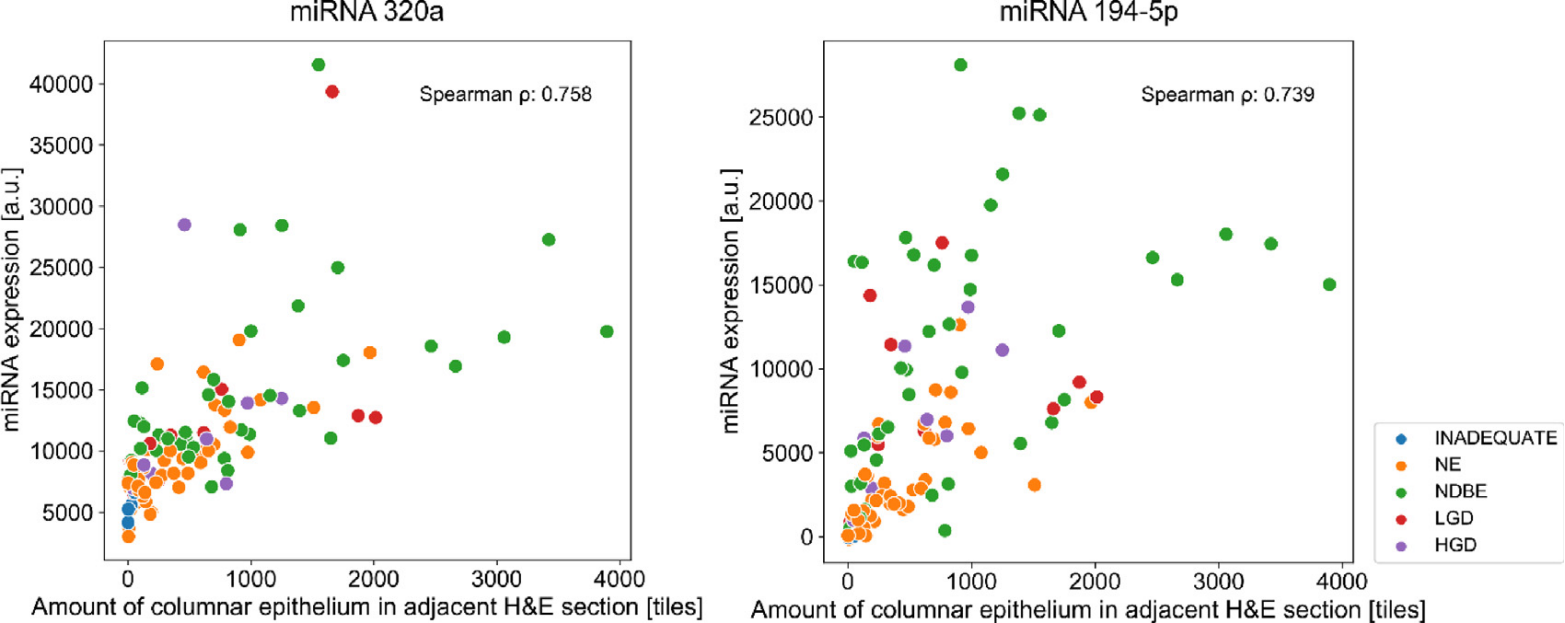
Correlation of CE amount and miRNA expression for markers with the highest Spearman ρ (rho) value. Cytosponge samples from Cohort 1. Inadequates, *n* = 3; NE, *n* = 46; NDBE, *n* = 47; LGD, *n* = 10; HGD, *n* = 10.

To explore the relationship between the type of epithelial content and miRNA expression, we quantified the amount of CE in contiguous H&E slides to the scrolls used for RNA extraction with a computational pathology tool. Ten miRNA targets analysed in Cohort 1 showed a positive correlation (ρ≥ 0.6) with the amount of CE in the H&E slides (Figure 5 for the top two markers and Supplementary Figure S4 for all 10) in a Spearman correlation. These were miR-320a (ρ = 0.76), miR-194–5p (0.74), miR-106b-5p (0.71), miR-30a-5p (0.70), miR-192–5p (0.68), miR-29c-3p (0.65), miR-22–3p (0.67), miR-21–5p (0.61), miR-28–5p (0.61), and miR-192–3p (0.60).

### miR-192–5p and miR-194–5p show a high sensitivity and specificity to detect presence of columnar epithelium in Cytosponge samples

To robustly explore the relationship between the presence of gastric cells and specific miRNAs, Cohort 2 was designed consisting of 72 inadequate and 67 adequate samples, both containing NE and NDBE cases (see Table 1 for details), and contiguous H&E slides were screened as above to quantify CE content. The expression of the four most informative miRs was assessed via qPCR: hsa-miR-192–3p, −192–5p, 194–5p, and −196a-5p. Three miRs (miR-192–5p, −194–5p and −196a-5p) were upregulated in measurements with four different platforms, including FirePlex™, microarray, nCounter, and qPCR (Supplementary Figure S5).^14^ The same three miRs showed to be upregulated in our diseased vs healthy test (Figure 3), the 4-group test (Supplementary Figure S3) and the BE vs NE differential analysis (Figure 4). miR-192–3p was added to the q-PCR test since it was also prominent in the latter three analyses as well as showing a positive correlation to the presence of CE (Supplementary Figure S4).

**Figure 6:**
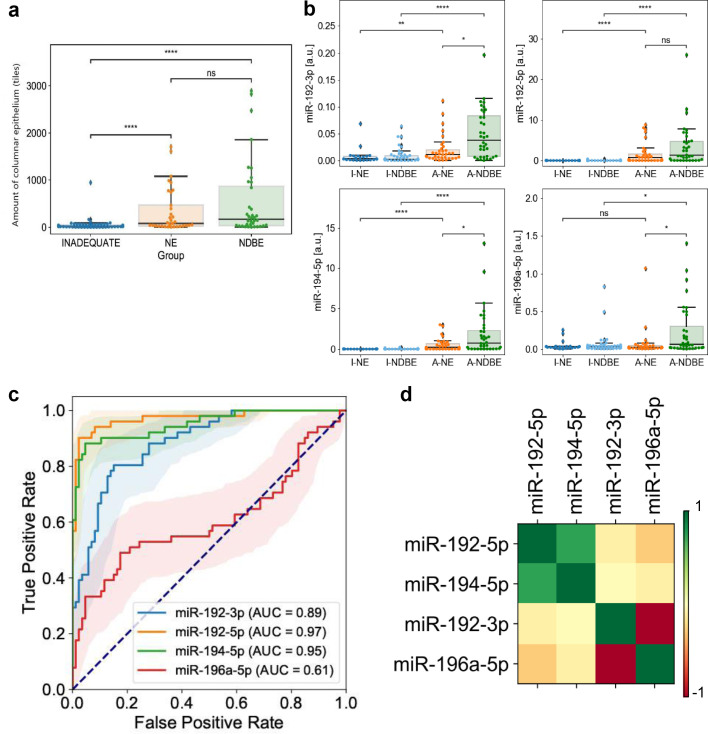
Cohort 2. **a**. CE content per sample and group done by means of number of tiles with presence of CE. The measurements were taken in the adjacent H&E slide to the one used for miRNA extraction (α=0.05 [Kruskall-Wallis]). Inadequate, *n* = 67. Adequate: NE, *n* = 36; NDBE, *n* = 36. **b**. Expression of the four test miRNAs in adequate (NE, *n* = 36 and NDBE, *n* = 36) and inadequate (NE, *n* = 24 and NDBE, *n* = 43) groups. The y axis is set for each marker individually as it shows 2^−(dCt)^ values (α=0.05 [Kruskall-Wallis]). **c**. ROC curve of four miRNA efficacy to determine presence of CE in the Cytosponge. Specificity is fixed at 95% for all markers. **d**. Clustered heat map from Cohort 2 data. The colour bar indicates correlation between displayed miRNAs from −1 to +1. While miR-192–5p and miR-194–5p behave similarly, miR-196a-5p behaves in an opposite way and miR-192–3p sits in the middle.

Quantitative computational pathology analysis of the H&E slides showed that while the average amount of CE for the NDBE group was higher than for the NE group, the difference was not statistically significant. Instead, statistically significant differences were found between adequate (either NE or NDBE) and inadequate samples (Figure 6A). qPCR experiments showed statistically significant differences between NE and NDBE cases within adequate samples for three of the four miRNAs analysed (Figure 6b), and all four miRNAs showed 2- to 3-fold changes between NDBE and NE groups. However, the fold changes in miRNA expression for miR-192–5p and −194–5p reached 98 and 60-fold respectively in adequate compared to inadequate samples. Contrary to that, miR-196a-5p was not differentially expressed between adequate and inadequate groups (Supplementary Figure S6). Rather, miR-196a-5p was upregulated in NDBE cases compared to NE in the adequate group, thus showing its potential for a true NDBE marker (Figure 6b). Moreover, a clustering analysis of the four miRNA expression patterns depicts the similarity between miR-192–5p and miR-194–5p and the distinctiveness of miR-196a-5p (Figure 6d).

A 5-fold cross-validation of a simple Random Forest model predicting sample adequacy based on the four miRNAs showed that two of the markers were able to identify presence of CE in miRNA from Cytosponges very reliably. Individually and at a specificity fixed at 95%, miR-192–3p, miR-192–5p, miR-194–5p and miR-196a-5p reached a sensitivity (and area under the curve, AUC; confidence intervals) of 41.18% (0.89; 0.82–0.94), 90.2% (0.97; 0.94–1.00), 84.31% (0.95; 0.91–0.98), and 25.49% (0.61; 0.51–0.71), respectively (Figure 6c).

Via molecular profiling, computational pathology, artificial intelligence, and pathway analysis, this study explores miRNA expression profiles as BE biomarkers in Cytosponge samples.

## Discussion

MicroRNAs are short, non-coding RNAs that regulate gene expression by binding to mRNA thus halting their translation to protein^27^ and can be key in neoplastic processes.^28^^,^^29^ The present study demonstrates that miRNA markers can be used to classify non-endoscopic cytology samples from the oesophagus. This type of sample contains cells from the entire oesophageal length, proximal stomach and oro-pharynx. Therefore, it is important to confirm the relationship between the biomarkers and the tissue of interest. Here we used artificial intelligence applied to a computational pathology tool to distinguish between miRNAs specific to the gastric columnar cells, which serve as a quality control metric for the sampling adequacy, compared with biomarkers specific to Barrett's metaplasia.

In this study, a custom Fireplex™ assay for multiplex discovery of miRNA targets was applied to oesophageal cytology samples. The platform uses probes that specifically capture amplified miRNA products in hydrogel particles which are in turn quantified by flow cytometry.^22^ In liver, brain and placenta tissues, a custom miRNA FirePlex™ panel previously showed ∼70% concordance with RNAseq data,^30^ while a further study showed a receiver operating characteristic curve (ROC-AUC) of 0.81 in detecting presence of pregnancy-specific miRNA in plasma.^31^ We report several upregulated miRs in Cytosponge samples from Barrett's with the initial multiplex miRNA expression analysis and 12 miRs with ≥2 fold changes in all three BE groups (NDBE, LGD and HGD) compared to NE. A potential limitation of this study is the fact that the sensitivity of the novel FirePlex technique to detect meaningful differential miRNA expression was unknown as the panel used was custom-made. Thus, no realistic power calculations could be performed when constructing Cohort 1. However, statistically significant differences were derived as demonstrated in Figs. 3 and 4, which suggests that the sample size was adequate.

The independent second cohort was designed to identify the tissue of origin of 4 miRNAs via qPCR analyses to potentially confirm them as a quality control tool for Cytosponge samples. The qPCR results confirmed the upregulation of the four miRs: miR-192–3p, −192–5p, −194–5p and 196a-5p in NDBE. This is in line with our previous publication, in which increased expression of the same four miRNAs was described in NDBE and dysplastic Cytosponges in comparison to healthy squamous tissue.^14^ Previously published studies had identified miR-192, miR-194 and miR-196a as BE markers in frozen or FFPE biopsies^18^^,^^32^, ^33^, ^34^, ^35^, ^36^, ^37^, ^38^, ^39^ as well as in plasma or serum for BE, EAC or ESCC^40^, ^41^, ^42^ and in other cancers.^43^^,^^44^ However, these studies did not include healthy gastric cardia samples in their cohorts except for one^38^ and thus the expression of the miRs in relevant adjacent tissues was not assessed.

An assessment of the amount of columnar epithelium (CE) in Cohort 1 showed a positive correlation (ρ≥ 0.6) between the expression of 10 miRNAs and the quantity of CE content. Cohort 2 showed that there were statistically significant differences in 3/4 analysed miRs between adequate and inadequate samples (those that do not contain any CE cells in the pathology slide). Further, a ROC analysis for the four test miRs showed that miR-192–5p and miR-194–5p can accurately determine the presence of CE with areas under the curve of 0.97 and 0.95 respectively. Thus, we deduce that miR-192–3p, miR-192–5p and miR-194–5p mark the presence of columnar material of the distal oesophagus, so they would be suitable as markers for adequate sampling, while miR-196a-5p is a specific BE marker.

The results from a pathway analysis of 17 miRs align with these hypotheses, where miR-196a-5p was linked to mRNAs involved in a glandular phenotype. In this analysis a further 6/17 miRs were associated to neoplastic or cell cycle processes (Table 2). In particular, genotoxic stress has been described as a p53-dependant promoter of the miR-192/215 axis^45^^,^^46^ and Fassan *et al*. describe a role for miR-192 and −215 in BE disease progression as well as the progressive upregulation of miR-194 from normal squamous epithelium to intestinal metaplasia.^35^ The marker could be playing an important role via the hedgehog pathway regulation. It is known that miR-194–5p targets the suppressor of fused homologue (SUFU) transcript, which is a Sonic Hedgehog repressor. This may help explain the elevated expression levels of Sonic Hedgehog proteins in BE as well as being a key element in the replacement of squamous by CE.^47^ Of note, as miR-194–5p is already upregulated in CE together with miR-215,^18^^,^^48^ this may suggest that gastric cardia is ontologically related to BE. In line with this, we saw how miR-194–5p displayed a high accuracy in identifying Cytosponge samples with CE.

Dysplasia-exclusive miRNA markers have proven to be elusive due to the focal nature of the lesions and molecular heterogeneity within the precancerous stage. In this study, several miRs were upregulated across the NDBE group through to dysplastic cases. These were miR-21–5p, −25–3p, −31–3p, −181b-5p, 192–5p, 194–5p, and −215–5p. In particular, miR-21–5p is a well-known cancer marker and it is thought to be involved in metastasis and apoptosis. Together with miR-25–3p,^45^ they are upregulated in liquid biopsies of EAC patients compared to healthy individuals.^49^ miR-31 is thought to be a progression marker via Wnt signalling, as it directly targets the Wnt inhibitor Dickkopf-related protein 1.^47^ Another well-described oncogenic miR is miR-181b-5p, which has been shown to play a role in oesophageal squamous cell carcinoma (ESCC),^50^ non-small cell lung cancer,^51^ triple-negative breast cancer^52^ and acute myeloid leukaemia^53^ among others. We observed the upregulation of miR-192 and miR-194 to be maintained through to the HGD group in Cohort 1. Interestingly, most of the miRs in this study that maintained their upregulation to dysplasia showed a pathway enrichment in neoplastic processes, be it direct or indirect.

Newer technologies such as single cell sequencing are now technically possible and can identify the cell of origin of certain omics signals. However, these single-cell approaches are expensive and not feasible for large clinical biomarker studies. Here we show how combining bulk-omics with computational pathology can help decipher the source of a biomarker signal. We have previously shown that being able to detect inadequate samples to obtain a repeated Cytosponge test from the patient increases the sensitivity of the Cytosponge-TFF3 test from approximately 80% to 90–92%.^14^^,^^16^ Here, the use of miRNAs in a molecular assay format has the potential to provide a quantitative read-out at scale with the ability to provide a QC for adequate sampling as well as to report on the presence of BE.

In summary, these results show how characterization of sampled material using different biomarker platforms and orthogonal methods can uncover the biological basis for the observed signal and avoid the introduction of bias in biomarker discovery. Furthermore, miRNA analyses have promise for use with Cytosponge and future work is required to test this method prospectively and identify dysplasia-specific markers.

## Declaration of interests

Izzuddin Diwan, Conor Rafferty and Elnaz Atabakhsh have been or are employees of Abcam. Abcam provided a subsidy to the Fitzgerald Lab (Cassandra Kosmidou, Neus Masque-Soler, Xiaodun Li and Rebecca C. Fitzgerald) on FirePlex™ reagents for the project.

Rebecca C. Fitzgerald (RCF) is named on patents related to Cytosponge and related assays which have been licensed by the Medical Research Council to Covidien GI Solutions (now Medtronic), however this was an academic study with no involvement from Medtronic. RCF has previously received fees from Medtronic for presentations and is also a co-founder and shareholder of an Early Detection company Cyted Ltd.. RCF is a Senior Associate Editor at the American Gastro Association journal Gastroenterology. Marcel Gehrung is an employee and shareholder of Cyted Ltd. All other authors declare no potential conflicts of interest.

## Appendix Supplementary materials

Image, application 1
