# Supplementary material for: Computational pathology aids derivation of microRNA biomarker signals from Cytosponge samples
Source: eBioMedicine. 2022 Jan 17;76:103814. doi: 10.1016/j.ebiom.2022.103814 (PMC8883000; doi:10.1016/j.ebiom.2022.103814)

**Supplementary methods**

FirePlex sample processing method

A Lysis Mix was made with Protease Mix and Digest Buffer from FirePlex’s miRNA Assay kit V2. For a negative sample control, a tube containing only water was included in the sample group and processed equally albeit without receiving Lysis Mix. This was later used as background subtraction for the rest of the plate. An FFPE scroll and a standard RNA in solution were supplied by the manufacturer and added to the cohort as extraction and assay positive controls. The positive and negative controls were always included as duplicates. All the other samples and controls received 250 μl of Lysis Mix and were incubated at 60°C with a 750 rpm shaking function for 45 minutes. Samples were pulsed vortexed after the first 10 minutes of incubation to ensure full submersion of the scrolls. After the remaining 35 minutes of incubation the tubes were vortexed and centrifuged at 2000 g for 1 minute, incubated for 2 minutes at room temperature (RT) and for a further 5 minutes on ice. The resulting liquid containing the extracted miRNAs was transferred to a filter plate provided with the kit. A catch plate was placed into the vacuum manifold, with the filter plate on top. Upon suction of the pump the samples were filtered and collected in the catch plate, and the filter plate was discarded. The samples were then stored at -20°C or processed further.

To prepare the hydrogel particles, 2 ml of 1X Rinse Buffer A was mixed with 2 ml of Panel A and B respectively. From the diluted panels, 70 μl of hydrogel particles from Panel A were applied to one half of a clean filter plate. The same was done for Panel B on the other half of the filter plate. To mitigate plate variability effects, panel A and panel B were applied in the left and right half of 96-well plates respectively, ensuring that each sample was analyzed in the same plate with both panels. Each hydrogel particle contains a high number of oligonucleotides specific for one miRNA and at its two ends has a unique fluorescence pattern. Using the vacuum manifold, the liquid in the mix was removed and the underside of the filter plate was blotted with lint-free tissue. Twenty-five microliters of Hybridization Buffer were added to each well of the filter plate, followed by 25 μl of the eluted sample from the catch plate, thus exposing the oligonucleotides in the hydrogel particles to the extracted miRNA. A standard RNA sample was added in the sample group at this point as a positive control. The filter plate was covered with a plastic lid and incubated at 37°C for 60 minutes at 750 rpm to allow the miRNA samples to hybridize with the specific oligonucleotides present in the hydrogel particles. The filter plate was rinsed twice with 165 μl of 1 X Rinse Buffer A per well, removing the liquid with the use of the vacuum manifold each time. The plate was blotted and 75 μl of 1 X Labeling Mix was applied to each well to end-label the hybridized miRNAs bound to the hydrogel particles. The filter plate was covered with a plastic lid and incubated at RT for 60 minutes at 750 rpm.

The following process allows for the recovery of the hybridized and labelled miRNA sample. The wells of the filter plate were rinsed and vacuumed twice with 165 μl of 1 X Rinse Buffer B and once with 1 X Rinse Buffer A, to get rid of unspecific hybridization, followed by a blotting after the last wash. One-hundred and ten microliters of RNase-free water were then applied to each well of the filter plate and it was sealed before being incubated at 55°C for 30 minutes while shaking, allowing the hybridized miRNA to melt-off from the probes within the hydrogel particles. Next, a clean catch plate was fitted into the bottom of the vacuum manifold with the filter plate on top, to transfer the melt-off product to the catch plate by suction. The filter plate containing the hydrogel particles was then stored in the fridge with 165 μl of 1 X Rinse Buffer A in each well to prevent dehydration of the particles.

A total of 30 μl of the melt-off eluent from the catch plate was then transferred to a PCR plate and 20 μl of the PCR master mix was added to each well. A PCR with universal primers containing a tag compatible with the Reporter Mix followed with 1 cycle at 93°C for 15 seconds; then 32 cycles of 93°C for 5 seconds, 57°C for 30 seconds and 68°C for 60 seconds; followed by 1 cycle at 68°C for 5 minutes, 1 cycle at 94°C for 4 minutes and a cooling off stage at 4°C.

When the PCR was finished, the filter plate containing the hydrogel particles was recovered from the fridge and its 1X Rinse Buffer A was removed by suction. A total of 60μl Hybridization Buffer was added to each well as well as 20μl of the resulting PCR reaction, to each corresponding well. This allowed the amplified product to re-bind to the hydrogel oligonucleotides. The plate was incubated for 30 minutes at 37°C and shaking at 750 rpm. Then, the filter plate was rinsed and vacuumed twice with 165 μl of 1 X Rinse Buffer B and once with 1 X Rinse Buffer A, the underside blotted. Having captured the amplicons, 75 μl of Reporter Mix was added to each well of the plate and left at RT and 750 rpm for 15 minutes to bind. A final two rinses with 1 X Rinse Buffer A were applied, with a final lint-free tissue blotting. One-hundred and ninety μl of 1 X PBS was added to each well of the Filter plate to allow for scanning on an LSRII (BD, Franklin Lakes, USA) or a Guava (Luminex, Austin, USA) flow cytometer.

Once the assay plates were processed, the .fcs files were loaded to the FirePlex Analysis Workbench software. A geometric mean normalization was applied across the plate and the two datasets from Panel A and Panel B were joined in a single dataset with all 110 targets. Once the data for each plate was generated, the plates processed with the same cytometer were merged by choosing the “combining experiments” option in the Workbench software. The combined data set was normalized with the geNorm algorithm. To integrate the two datasets from two different flow cytometers we established the required normalization factor. The data originating from the Guava cytometer was scaled up by a factor of 7, and all samples were put through one final geNorm process.

Quantitative PCR method

For the reverse transcription (RT-PCR) reaction, miScript II RT kit (QIAgen) with miScript HiSpec buffer was used. Once the RT-PCR was performed on a thermo cycler, the product was used in a 1:10 dilution on the quantitative PCR (qPCR). The QuantiNova SYBR green PCR kit and the universal reverse primer from miScript SYBR green kit (both from QIAgen) were used for the qPCR. The specific forward primers, which were used at 10 uM are listed in Supplementary Table ST5. The cycling reaction started at 95°C for 15 minutes, followed by 45 cycles of amplification steps of 95°C, 60°C and 72°C for 10 seconds each, and a melting curve of 95°C for 5 seconds, 65°C for 1 minute and 97°C, finished with a cooling step. Hsa-miR-103 and -191 were used for normalization consistent with previously published microarray data^12, 15, 22^.

**Supplementary data**

**Supplementary table ST1**. FirePlex panel design, including previous platforms used, designation to panel A or B and relevant literature per target.

**Cohort characteristics –** Cohort 1

**Supplementary figure S1**. Graphical representations of age, body mass index (BMI), waist-to-hip ratio, gender, and BE segment length in the three diagnostic groups of Cohort 1. Only statistically significant differences (α=0.05) are shown and whiskers represent 5/95 percentiles [two-tailed, unpaired t-tests].

Cohort 2

**Supplementary figure S2**. Graphical representations of age, body mass index (BMI), waist-to-hip ratio, gender, and BE segment length in NE or NDBE samples classified as Adequate or Inadequate subgroups. Only statistically significant differences (α=0.05) are shown and whiskers represent 5/95 percentiles [two-tailed, unpaired t-tests].


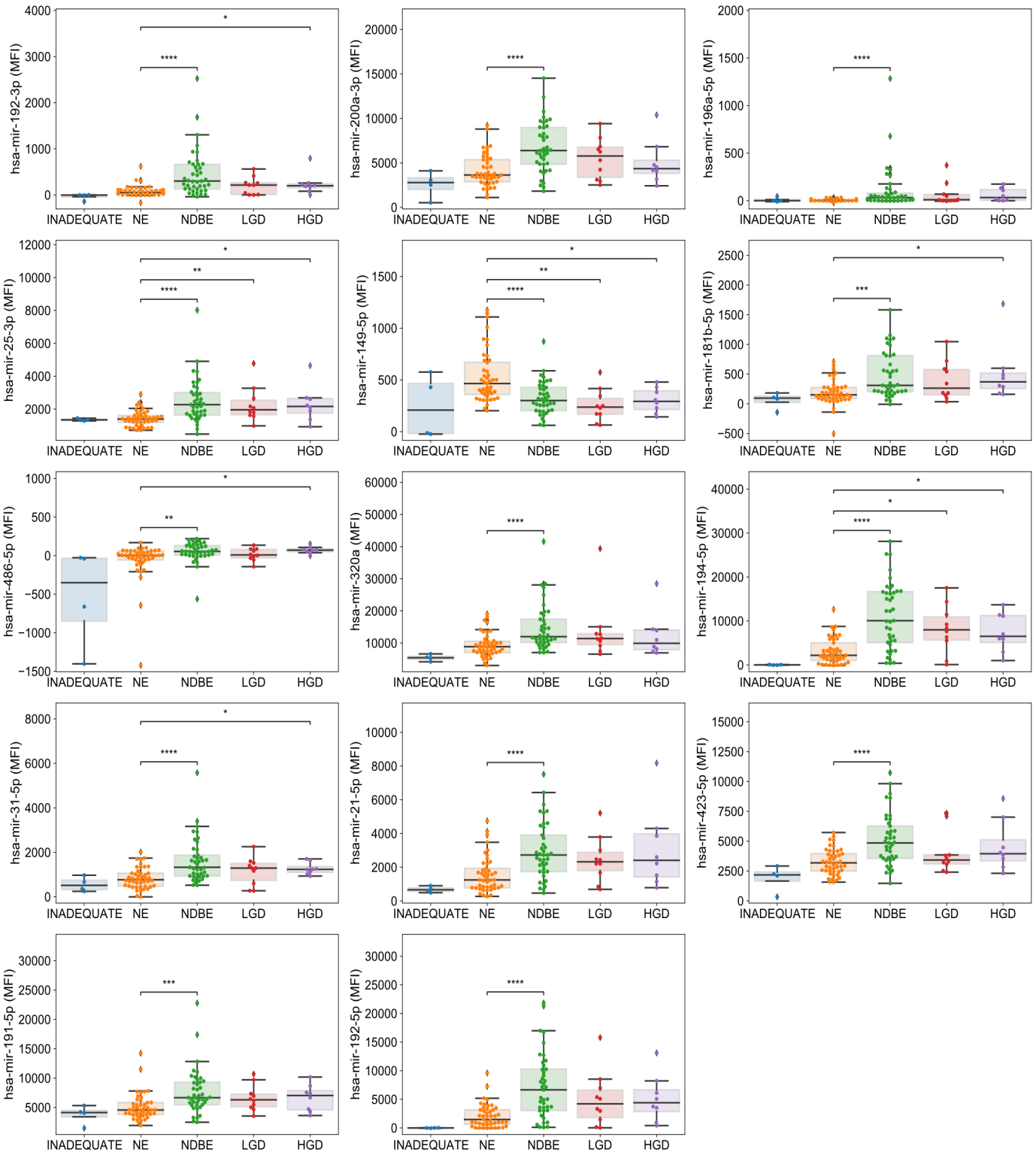


**Supplementary figure S3**. 4-group differential analysis [Kruskall-Wallis tests]. Fourteen markers show a differential expression in any of the four diagnostic groups: NE, NDBE, LGD and HGD. Only differences with statistical significance are shown.

**Supplementary Table ST4**. MiRNA targets with a median fold change ≥2 in NDBE, LGD or HGD compared to NE groups in FirePlex data. Highlighted in grey are the 12 targets with ≥2 in all three BE groups.


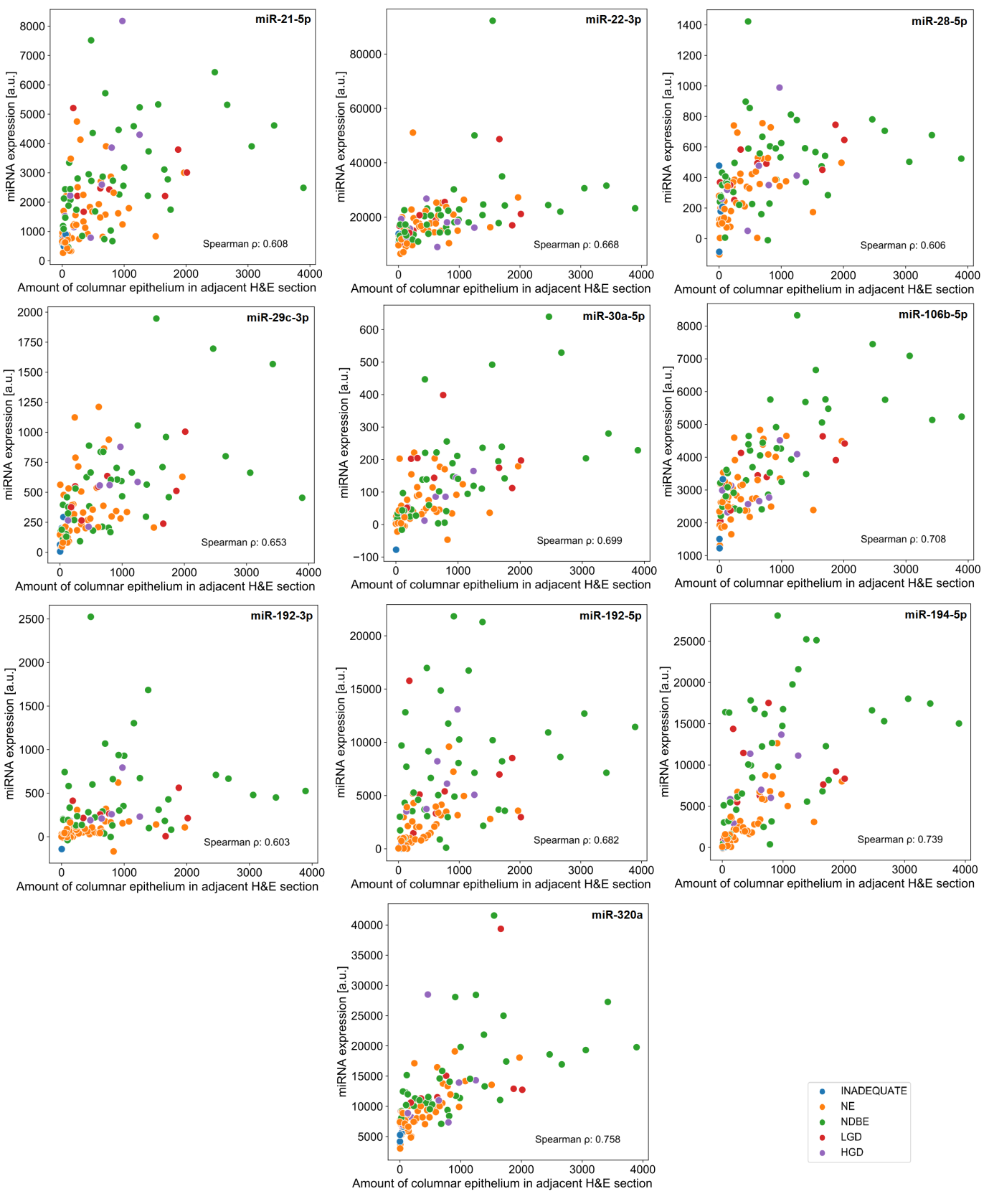


**Supplementary figure S4**. Correlation of CE amount and miRNA expression for rho ≥ 0.6 in samples from Cohort 1.

**Supplementary figure S5**. Cross-analysis comparison of up-regulated miRNA between BE and NE in this project and the study by Li *et al*. (Gastroenterology 2018;155:771–783). The four test miRNAs assessed in qPCR are highlighted in grey. *: miRNAs downregulated in BE compared to NE. The rest are all upregulated in BE.

| **miRNA target** | **Forward primer sequences** |
| --- | --- |
| hsa-miR-103b ctr | 5'-CAGCATTGTACAGGGCT-3' |
| hsa-miR-191-5p ctr | 5'-CGGAATCCCAAAAGCAG-3' |
| hsa-miR-192-3p | 5'-GCCAATTCCATAGGTCAC-3' |
| hsa-miR-192-5p | 5'-GACCTATGAATTGACAGC-3' |
| hsa-miR-194-5p | 5'-ACAGCAACTCCATGTGG-3' |
| hsa-miR-196a-5p | 5'-TAGGTAGTTTCATGTTGTTGG-3' |

**Supplementary table ST5**: Forward primers used in the qPCR.


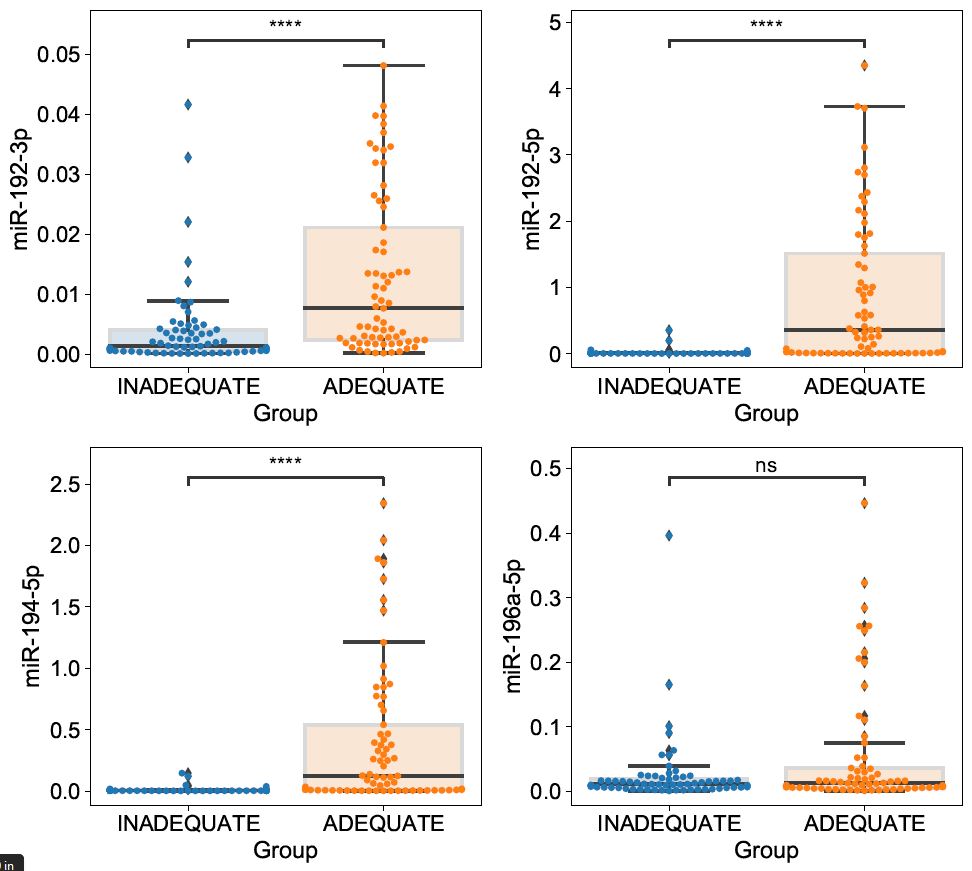


**Supplementary figure S6**: Expression of the 4 test miRNAs in adequate and inadequate groups in qPCR assays in Cohort 2. The y axis is set for each marker individually. All 3 markers with significant differences have p<0.0001 [two-tailed, unpaired t-tests].

**STRING database results**:

miR-192-3p


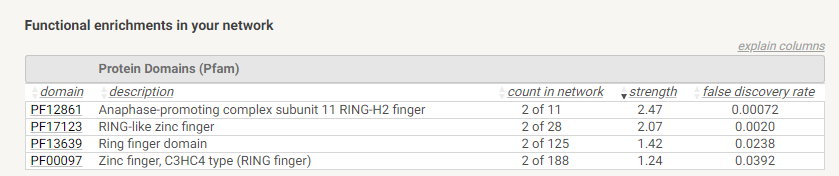


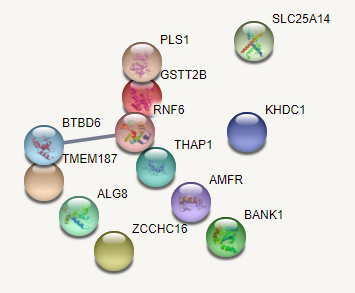


miR-192-5p


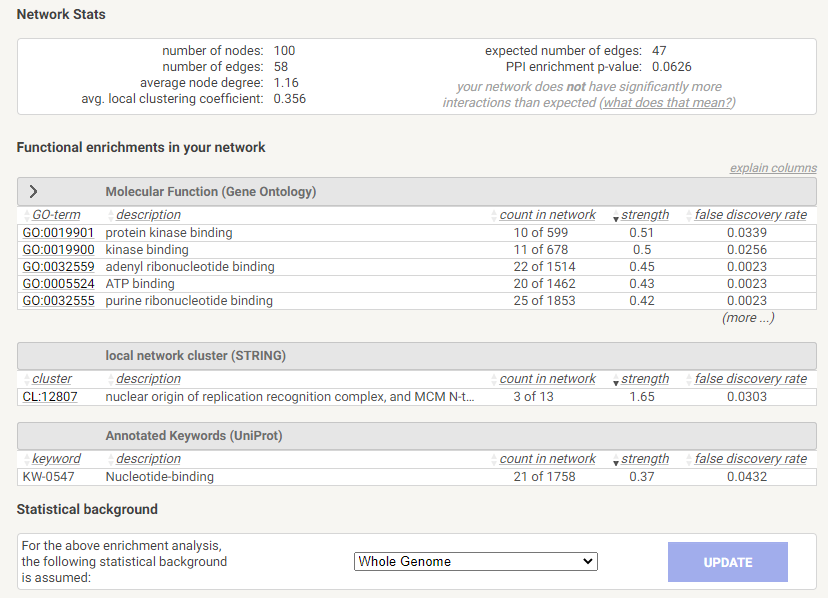


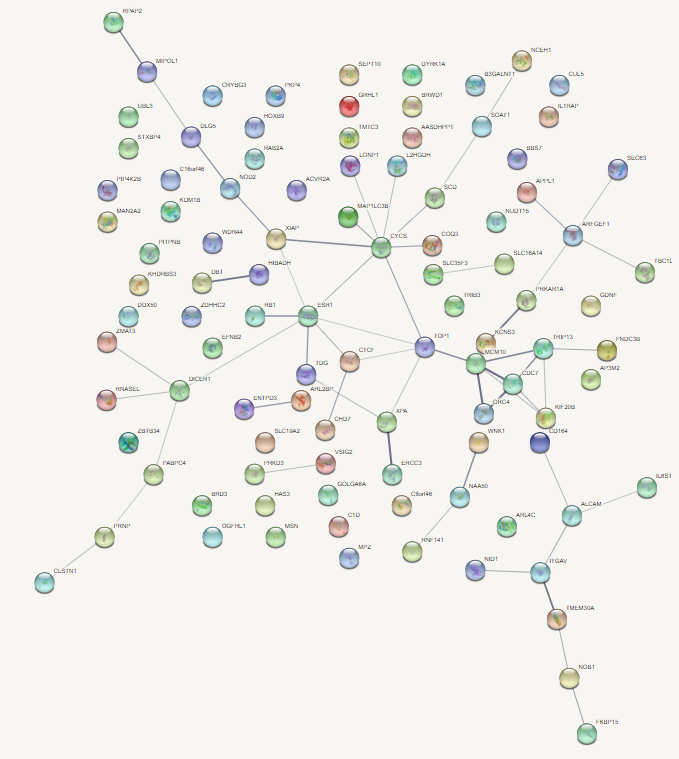


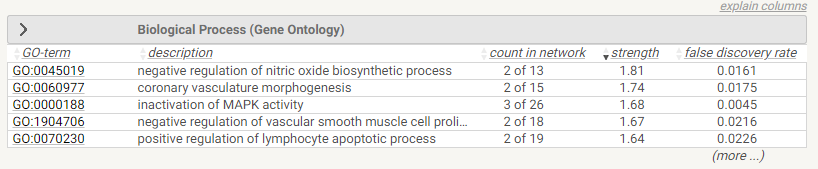
194-5p


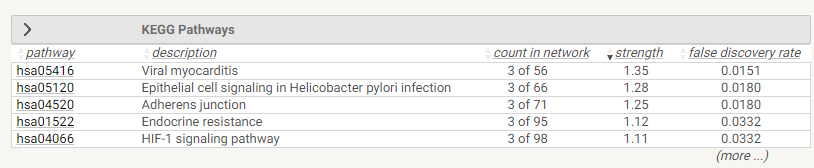


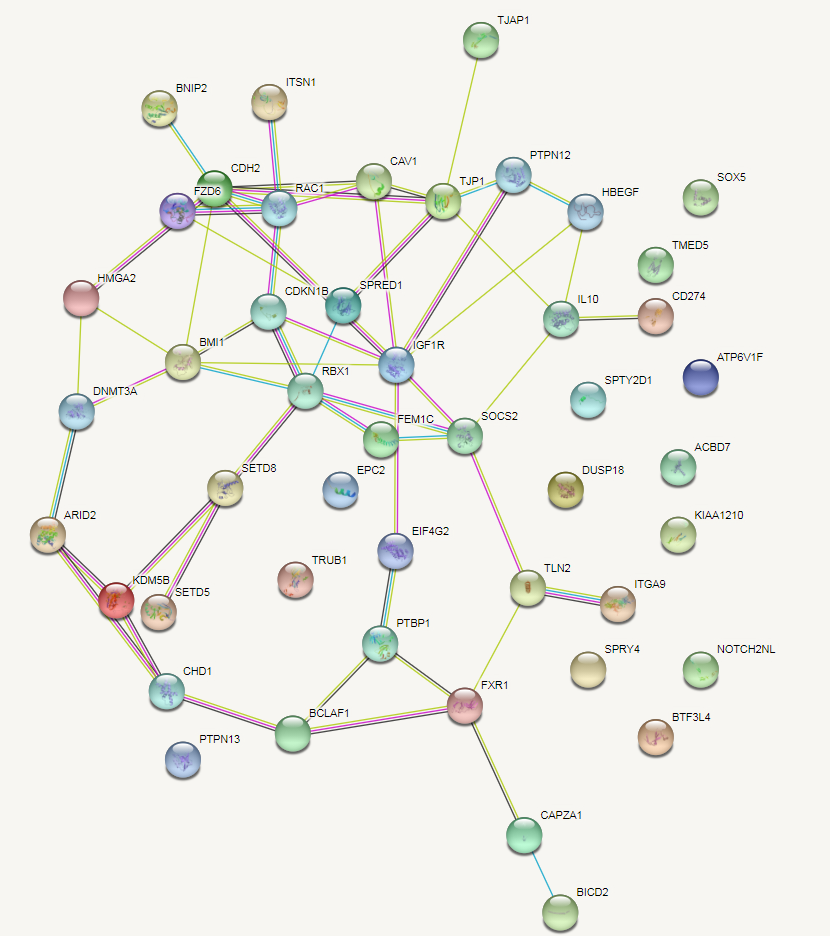


196a-5p


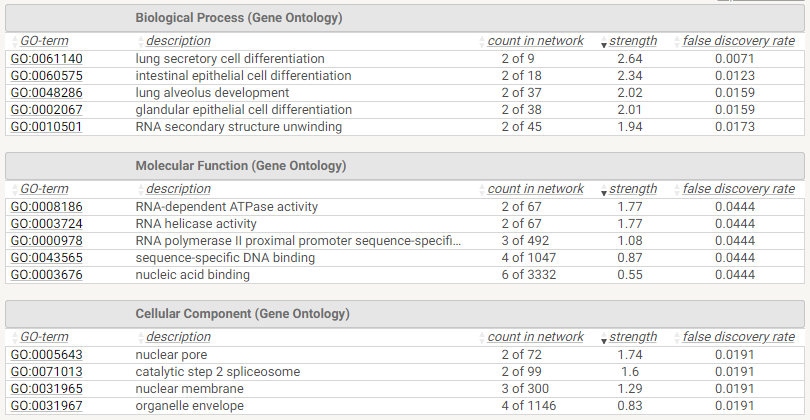


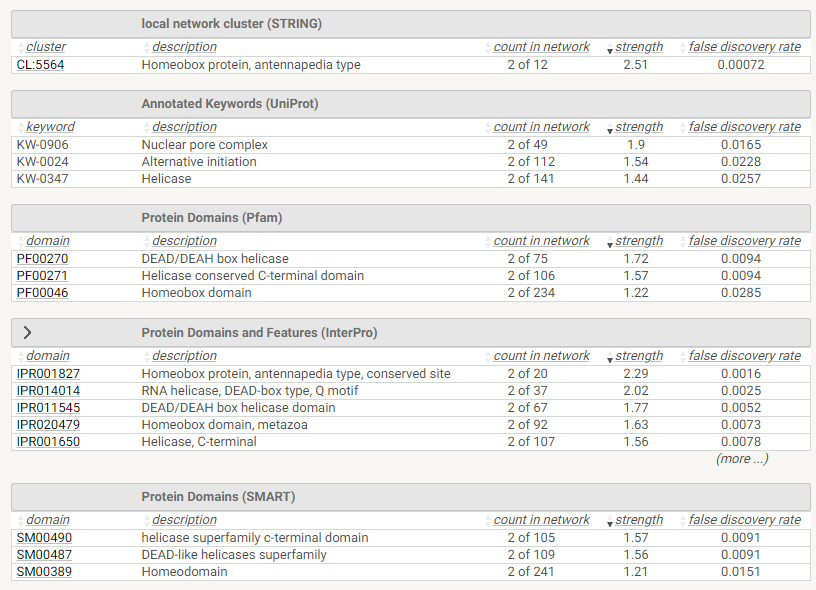


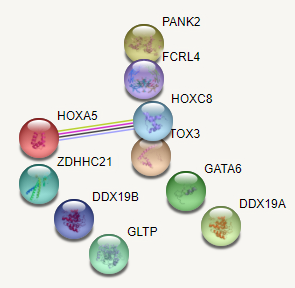


25-3p


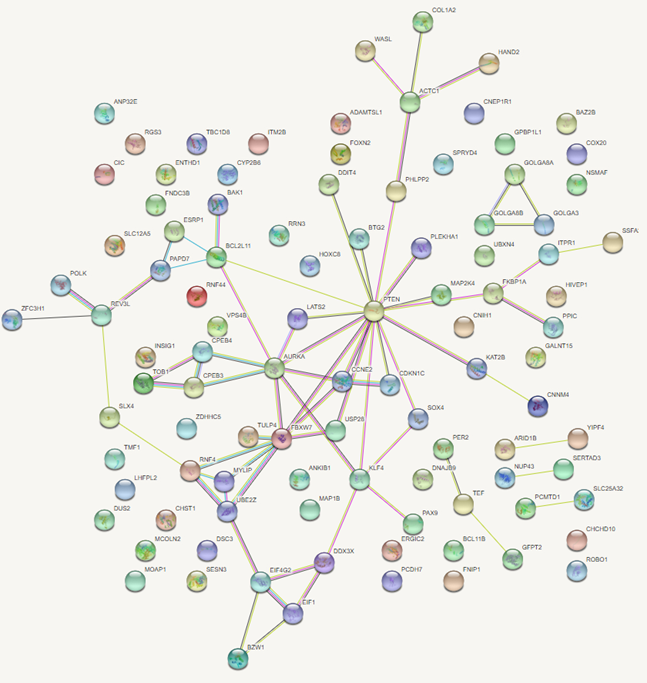

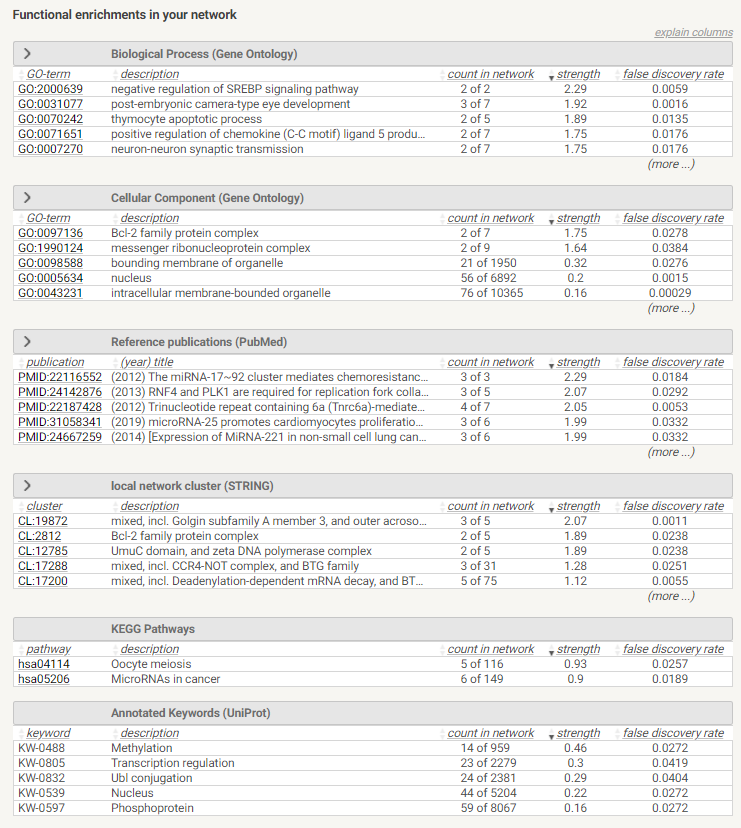


31-5p

No significant enrichment detected

181b-5p


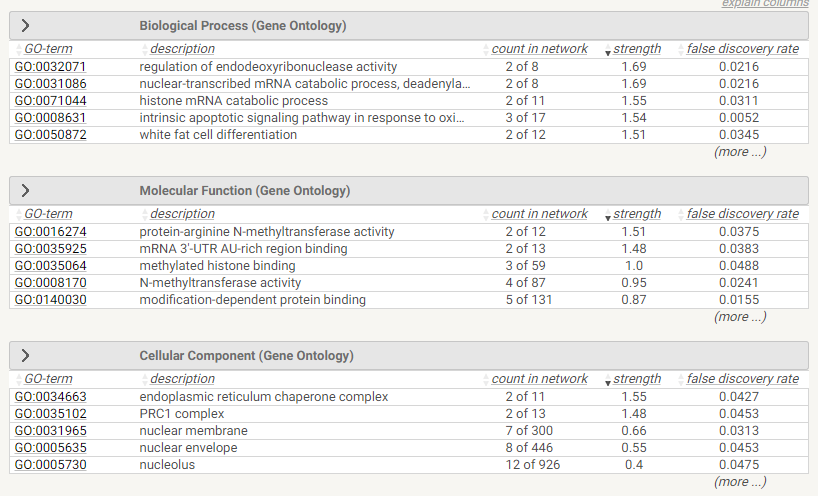


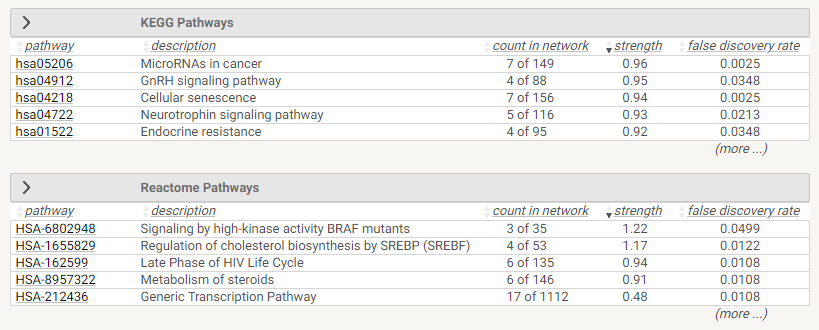


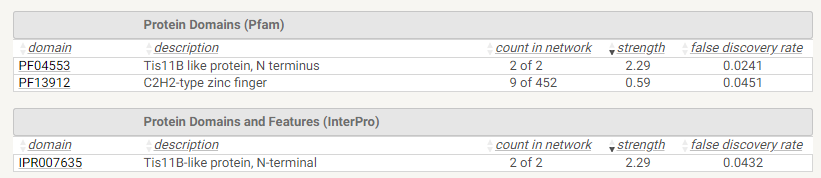


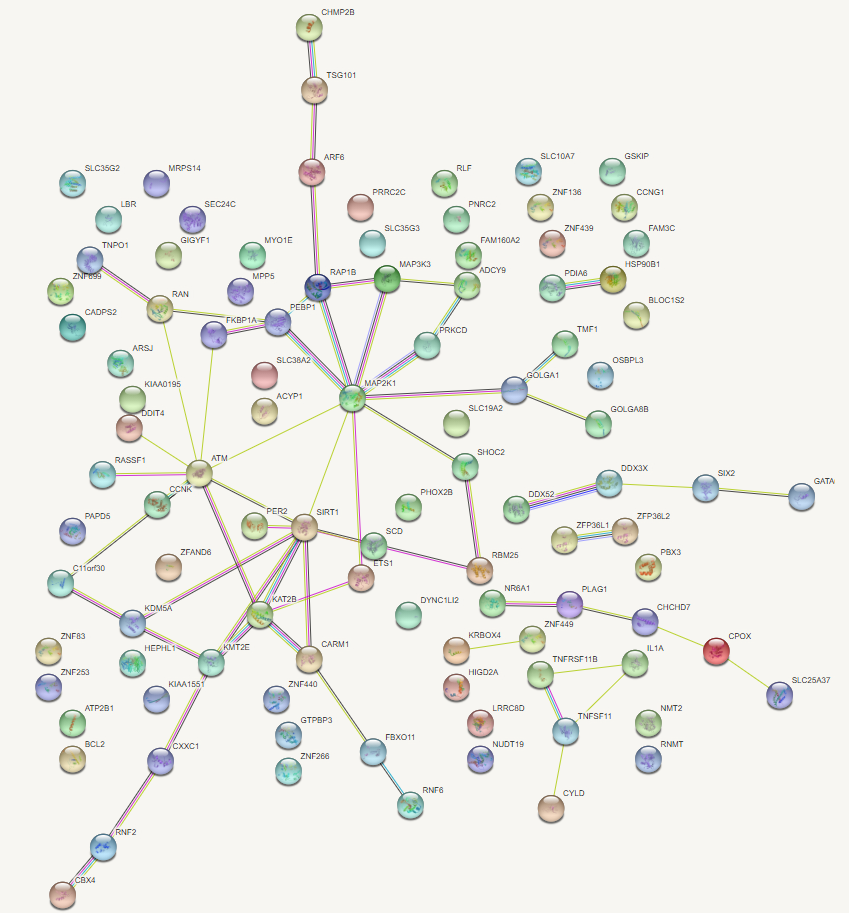


215-5p
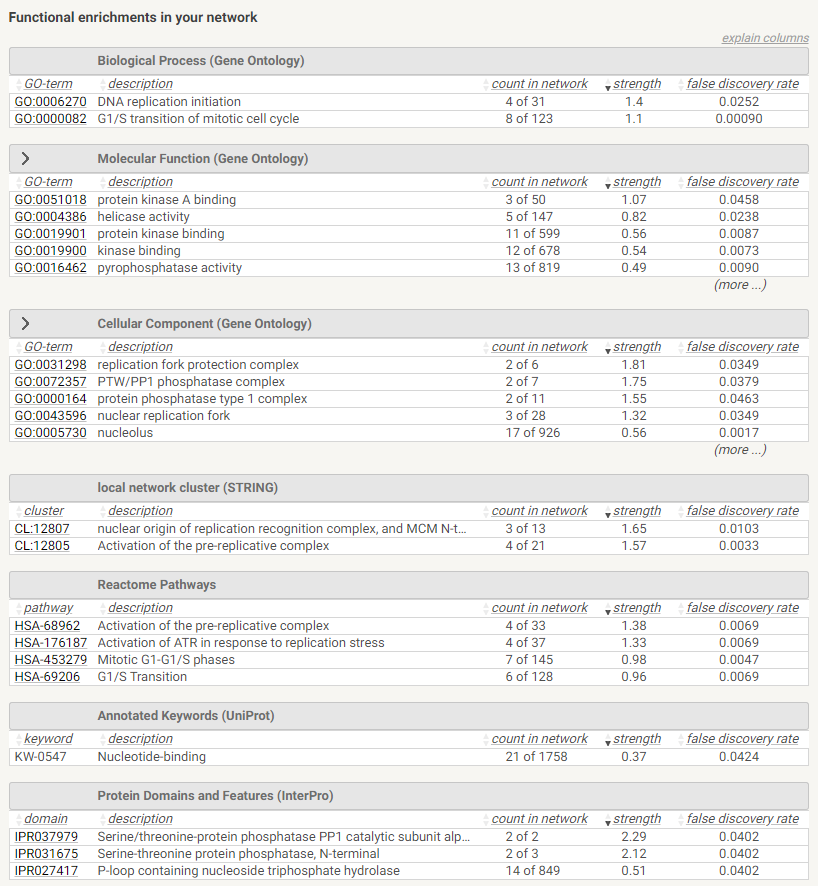


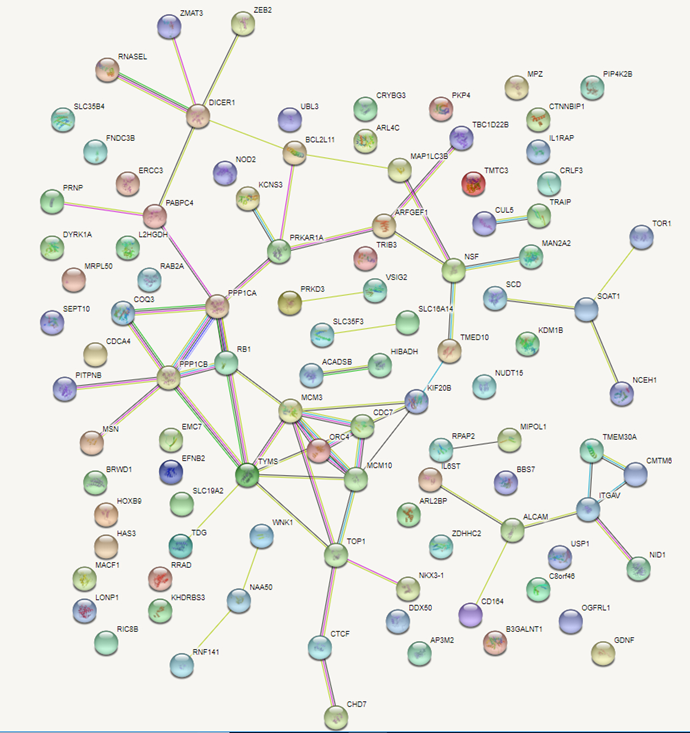


486-5p


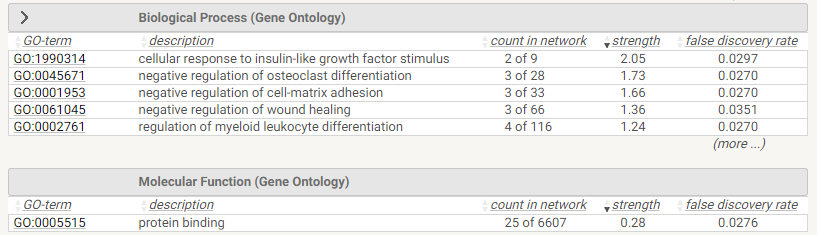


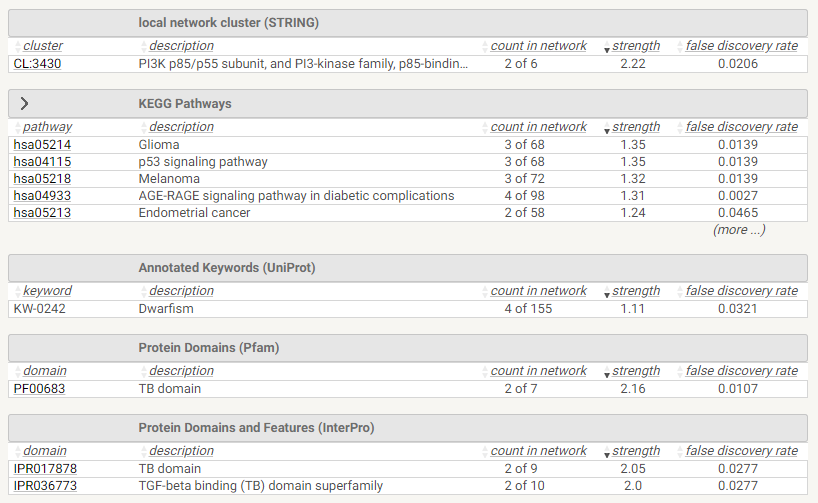


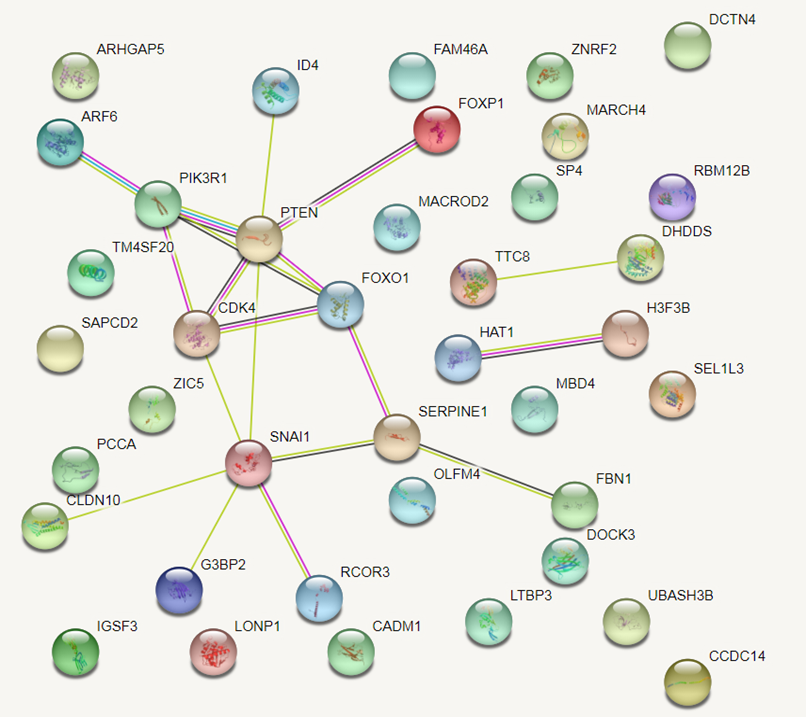

Supplement: Supplementary file 1 [file mmc1.docx]
